# Supplementary material for: Integrated microbiome and metabolome analysis reveals the interaction between intestinal flora and serum metabolites as potential biomarkers in hepatocellular carcinoma patients
Source: Front Cell Infect Microbiol. 2023 May 16;13:1170748. doi: 10.3389/fcimb.2023.1170748 (PMC10227431; doi:10.3389/fcimb.2023.1170748)
Supplement: Supplementary file 1 [file DataSheet_1.pdf]

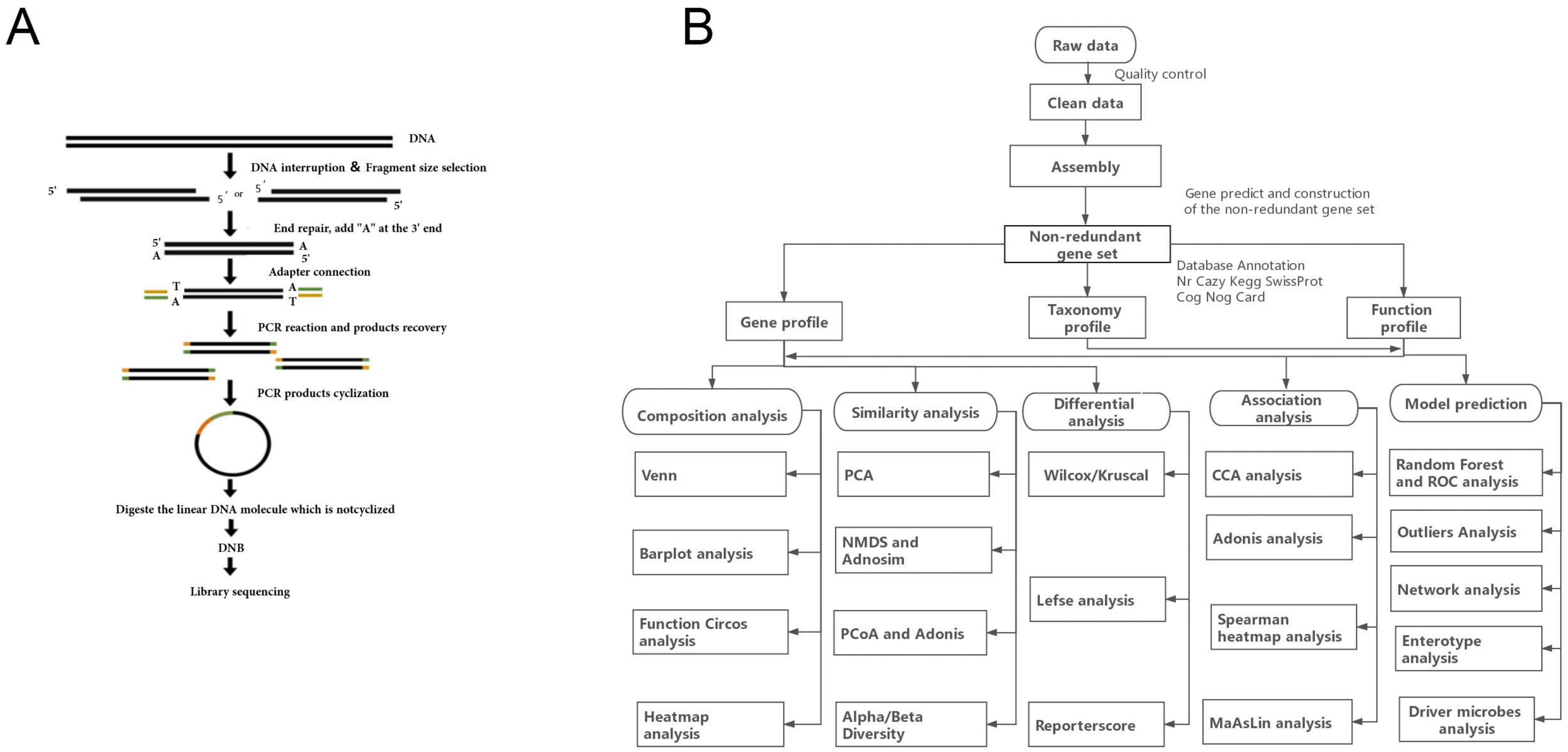

Supplementary figure S1. Experimental Workflow and Bioinformatic Analysis Workflow. (A) DNBSEQ library construction and sequencing. We set up strict quality control in each step in experimental workflow (such as sample processing, library prepareation, and sequencing). (B) The bioinformatic analysis workflow for metagenomics. High-quality reads assembly, gene prediction, gene de-redundancy and subsequent abundance construction, as well as species/function differential analysis.

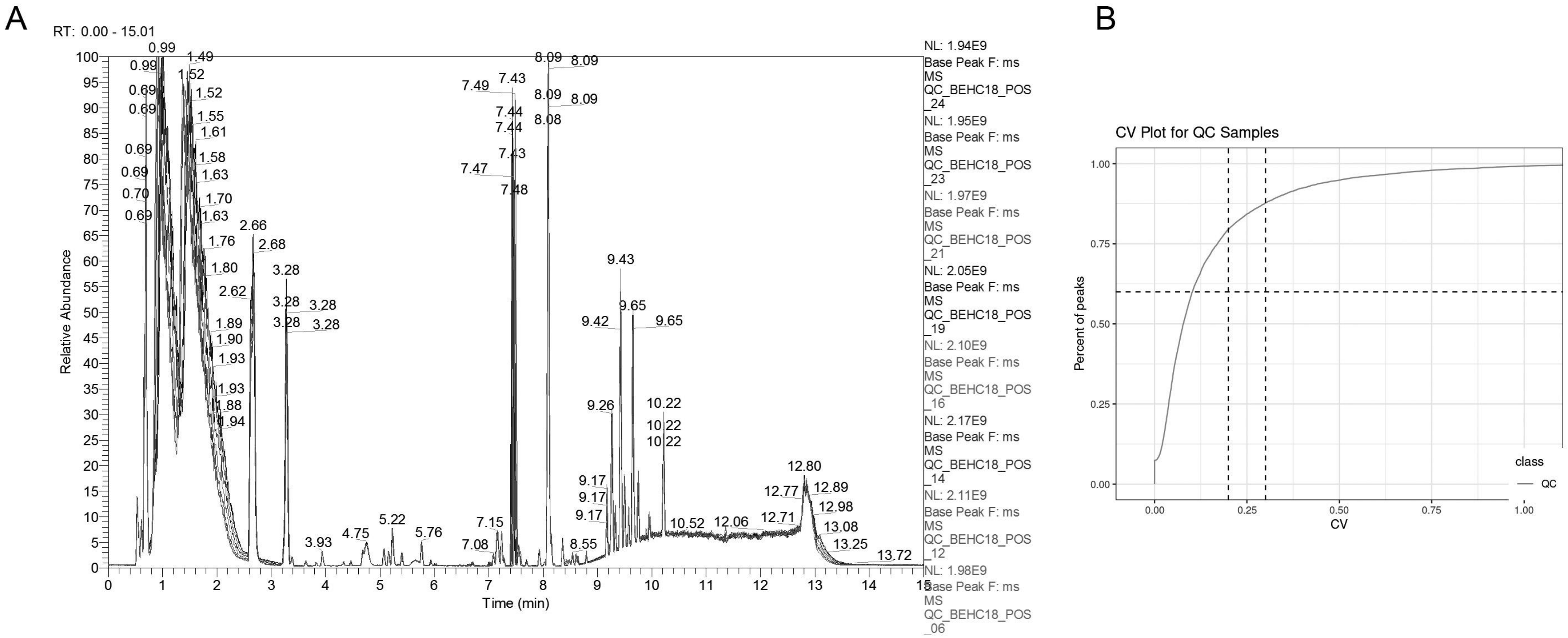

Supplementary figure S2. Data Quality Control.(A) BPC overlay of QC samples. BPC is a map that continuously depicts the intensity of the strongest ions in the mass spectrum at each time point.The BPC of all QC samples were overlapped, the spectrum overlap was good, and the retention time and peak response intensity fluctuated little, indicating that the instrument was in good condition and the signal was stable during the whole sample detection and analysis. (B) CV distribution of compounds in each sample. The two lines perpendicular to the X axis in the figure are 20%, 30% CV reference line, and the line parallel to the X axis is 60% of the reference line. Number of Compounds (CV<=30%): The number of compounds with a relative peak area CV of 30% or less in the QC sample. Ratio: The ratio of the number of compounds with a relative peak area CV less than or equal to 30% in the QC sample to the total number of compounds detected. Ratio >=60%, the data quality is qualified.

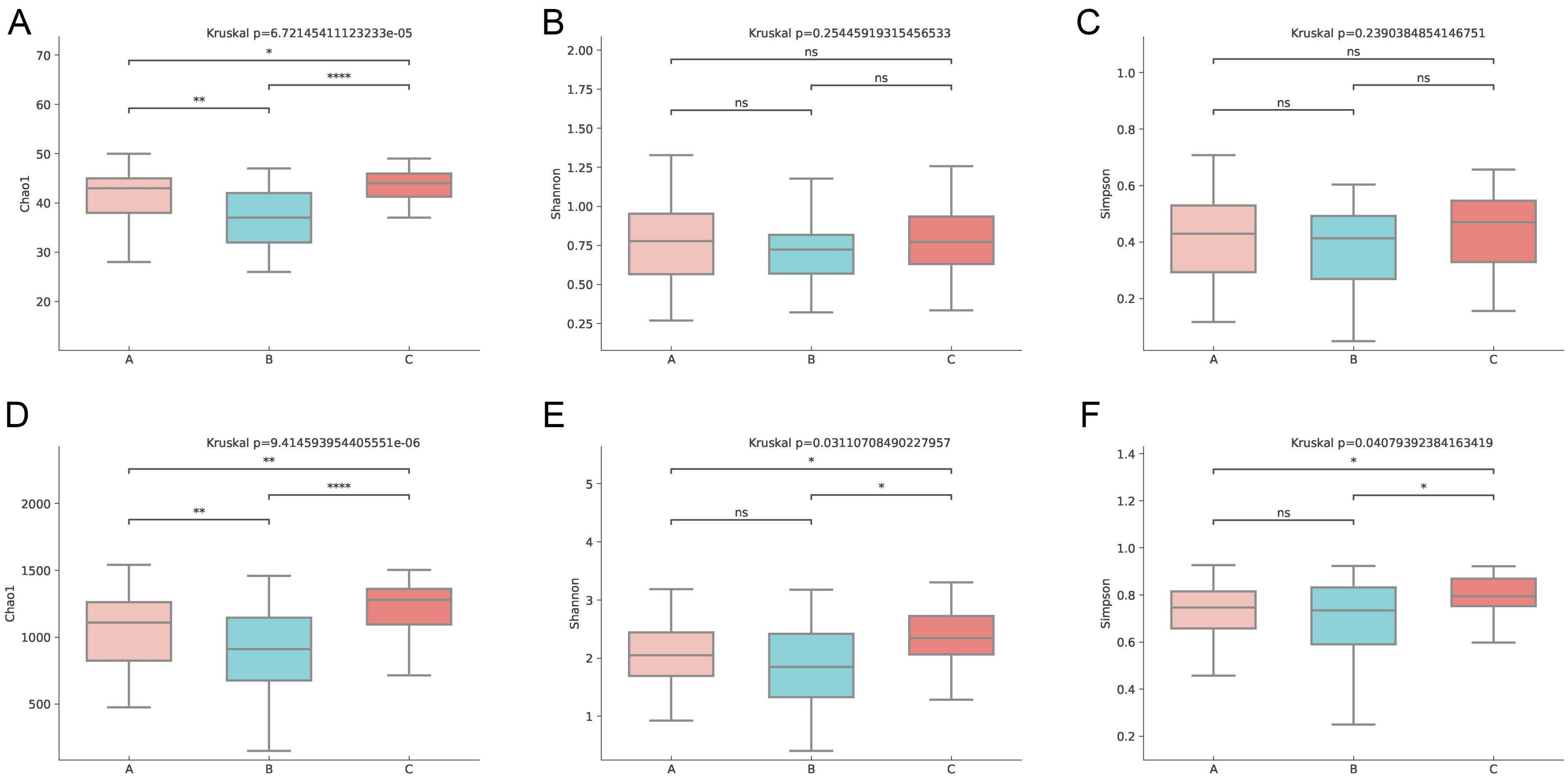

Supplementary figure S3. Alpha diversity at the phylum and genus level. *p < 0.05, **p < 0.01, ***p < 0.001

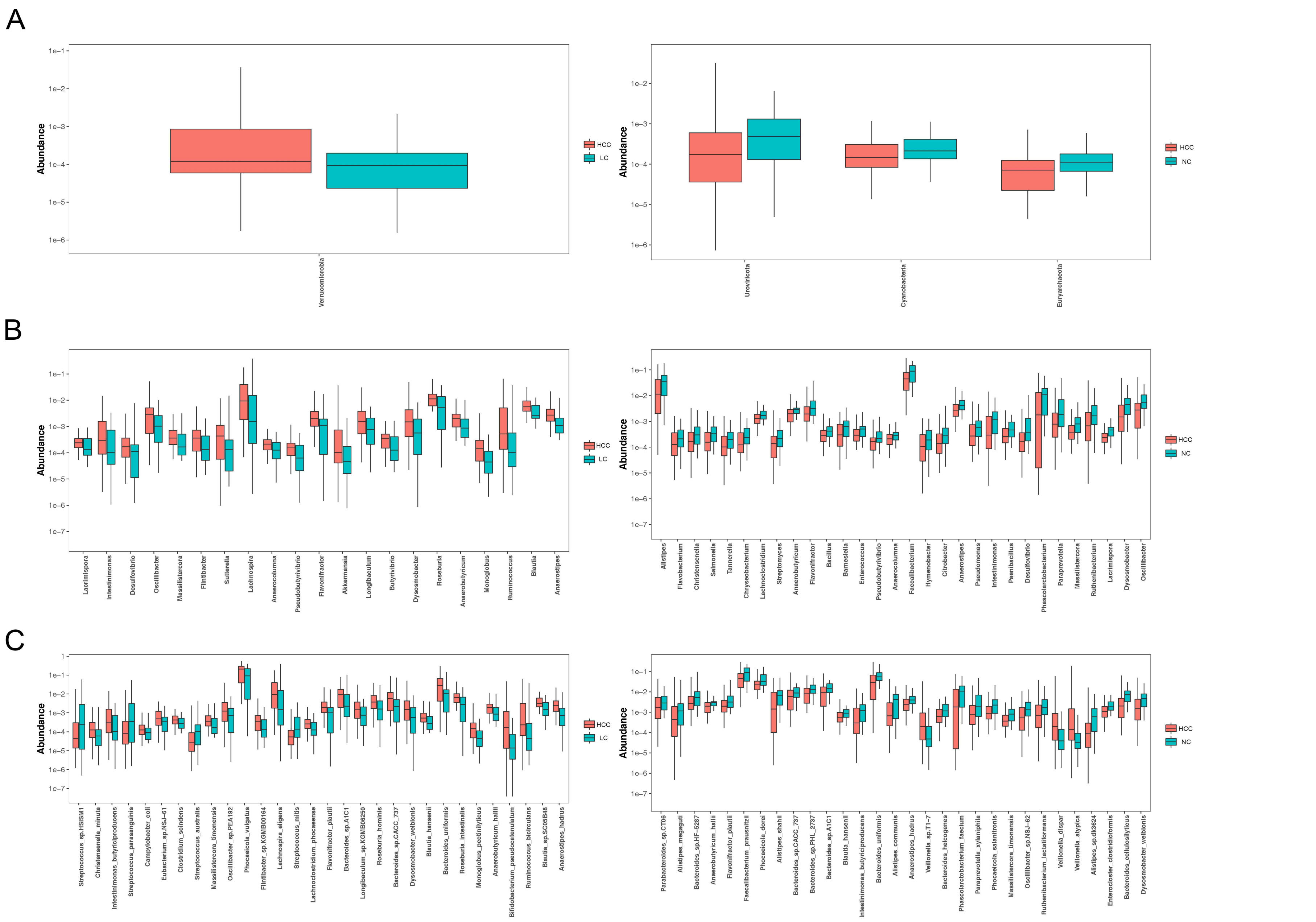

Supplementary figure S4. The Wilcoxon rank-sum test in HCC and non-HCC. (A) At the phylum level. (B) At the genus level. (C) At the phylum level. Only the relative abundance of top30 is given in the figure.

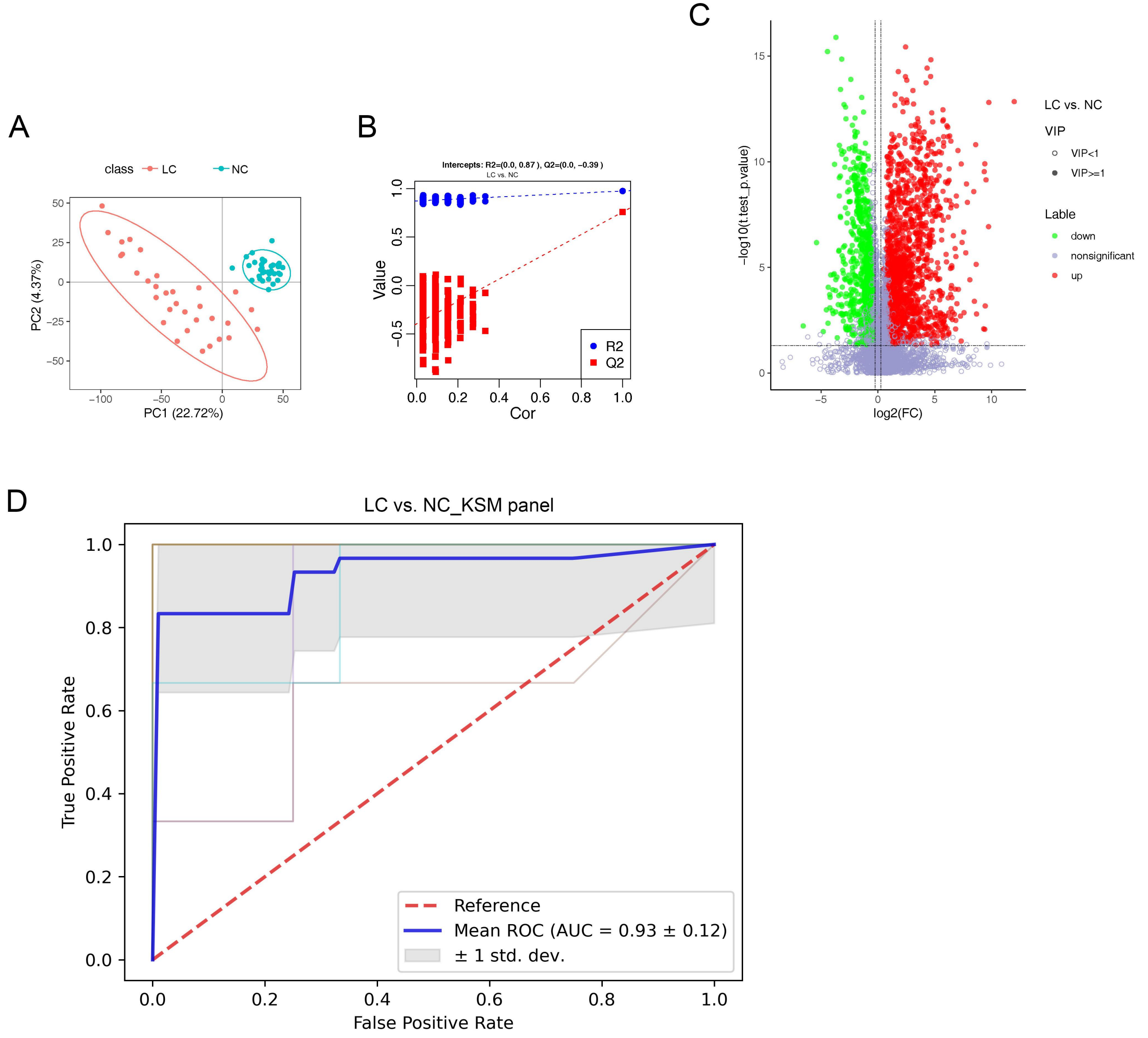

Supplementary figure S5. Serum metabolite changes in LC and NC. (A) PLS-DA shows the differences between LC and NC. The abscissa (PC1) and the ordinate (PC2) are the two main coordinates that explain the greatest difference between samples. The number is the score of the principal component, which represents the percentage of the explanation on overall variance of the specific pricipal component. The graph's points represent samples, and different colors represent various sample grouping information; similar samples are clustered together. (B) The two rightmost points in the figure are the actual R2Y and Q2 values of the model, and the remaining points are the R2Y and Q2 values obtained by randomly arranging the samples used. This result is mainly used to judge whether the model is overfit and the validity of the model. (C) Volcano Plot differ between LC and NC.Green is the down-regulated differential metabolite (labeled green), red is the up-regulated differential metabolite (labeled red), and metabolites without difference are labeled purple-gray. (D) The KSM panel had AUC values of 0.93±0.12 in LC versus NC?. KGM, key gut microbes; KSM, key serum metabolites; ROC, receiver operating characteristic; The abscissa of the ROC curve is the false positive rate, the ordinate is the true positive rate, the blue curve is the average curve after 10 folds, the AUC is the area under the curve, and the shaded region is the upper and lower 1 standard deviation.

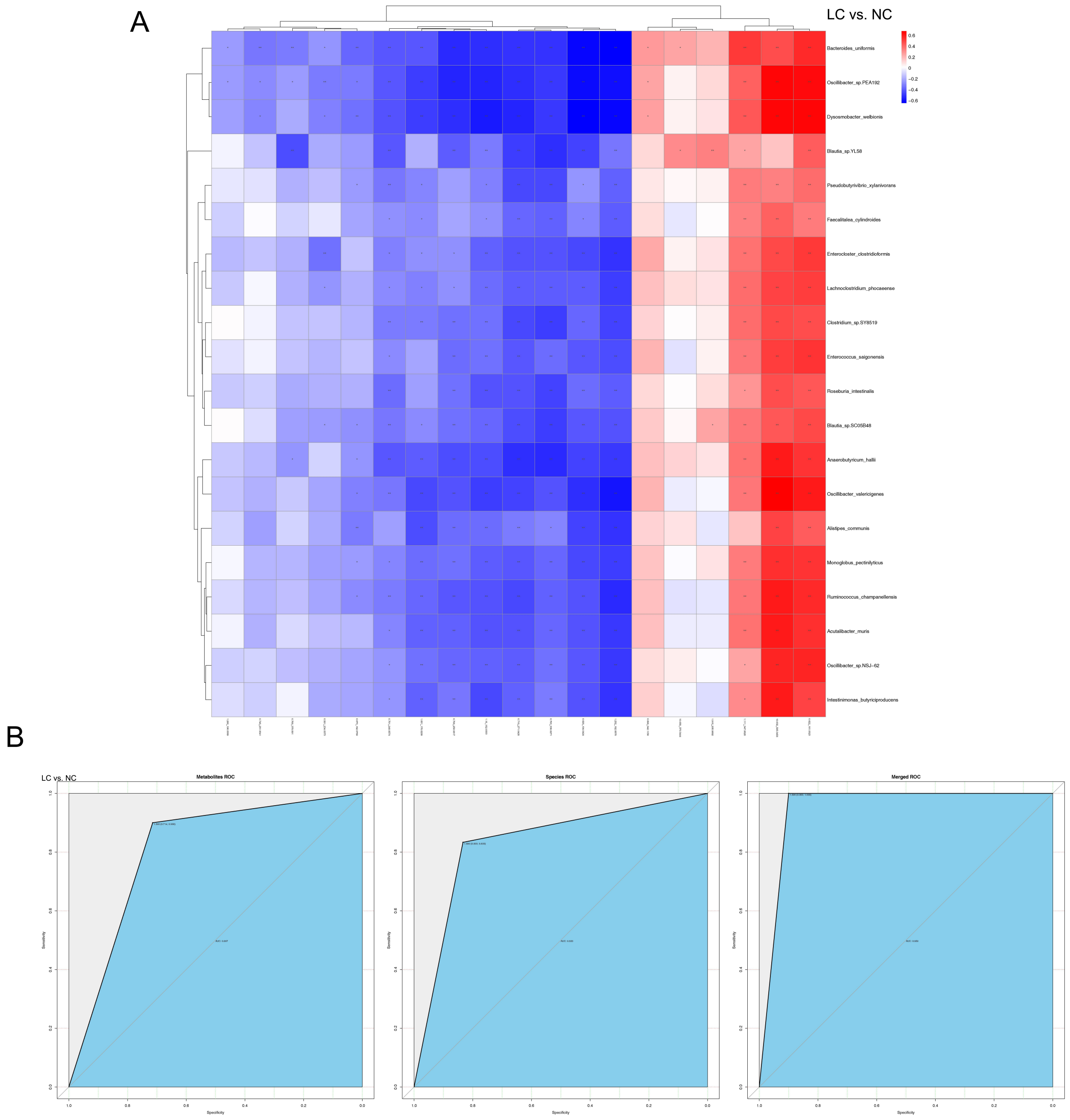

Supplementary figure S6. (A) The heat map of top 20 differential species and differential metabolites with the smallest p-values for every omics in LC vs. NC. Columns represent the differential metabolites and rows represent the differential species. The color blocks represent the correlation coefficient. The darker the color, the stronger the correlation between the different species and the different metabolites. Red represents positive correlation and blue represents negative correlation. * represents $p<0.05$, ** represents $p<0.01$. (B) The ROC curves of Random Forest analysis of species and metabolism biomarkers. Random forest ROC map of species and metabolome (ROC map of metabolome is on the left, ROC map of species is in the middle, and ROC map of species and metabolome is on the right).
